# Negative Pressure Wound Therapy With Flap Reconstruction for Extensive Soft Tissue Loss in the Foot: A Case Report

**DOI:** 10.7759/cureus.10116

**Published:** 2020-08-29

**Authors:** Kishore Vellingiri, Nagakumar J S, Deepak Hongaiah

**Affiliations:** 1 Orthopaedics, Sri Devaraj Urs Academy of Higher Education and Research, Kolar, IND; 2 Plastic Surgery, Sri Devaraj Urs Academy of Higher Education and Research, Kolar, IND

**Keywords:** negative pressure wound therapy

## Abstract

Negative pressure wound therapy (NPWT) can create the healing granulation tissue that will form a wound bed for the skin graft, thereby reducing the volume of the soft tissue defect. The application of uniform negative pressure, which is delivered by vacuum-assisted closure (VAC) therapy, induces a physical response (macrostrain) and a biological response (microstrain). The patient in the current case report presented with an alleged history of a road traffic accident, sustaining a crush injury to his right heel pad, resulting in an open comminuted fracture of the right calcaneum with bone loss. A total of seven days of NPWT was allowed. Negative pressure sponge dressing was then applied in this region and adhesive drapes were sealed. Once sealed, suction was set at the continuous pressure of -125 mm Hg. The authors noted that the benefits significantly outweigh the costs of the VAC system, making it an essential treatment option for patients similar to the one presented in this case report.

## Introduction

Negative pressure wound therapy (NPWT), also known as topical negative pressure therapy, has revolutionized the world of wound care. NPWT aids in forming granulation tissue, which serves as a wound bed for the skin graft and reduces the volume of soft tissue defects [1]. We report a case of a crush injury to the right heel pad, which caused an open comminuted fracture of the right calcaneum with bone loss. Initial debridement with regular dressing was done, which was associated with poor outcome. So a single set of seven days of vacuum-assisted closure (VAC) was tried for this patient after secondary debridement, which, in turn, turned out to be a game-changer for the patient with extensive soft tissue loss in the foot. VAC can thus potentially replace microsurgical soft tissue transfer, reduce the risk of infection, and aid in salvaging the limb.

The abstract of the case was accepted for paper presentation at the 53rd Annual Conference of Tamilnadu Orthopaedic Association (TNOACON) held from February 7 to 9, 2020, at Kingston Engineering College, Vellore, India. The abstract is scheduled to be published in the online supplement of the Tamilnadu Orthopaedic Association Journal.

## Case presentation

**Figure 1:**
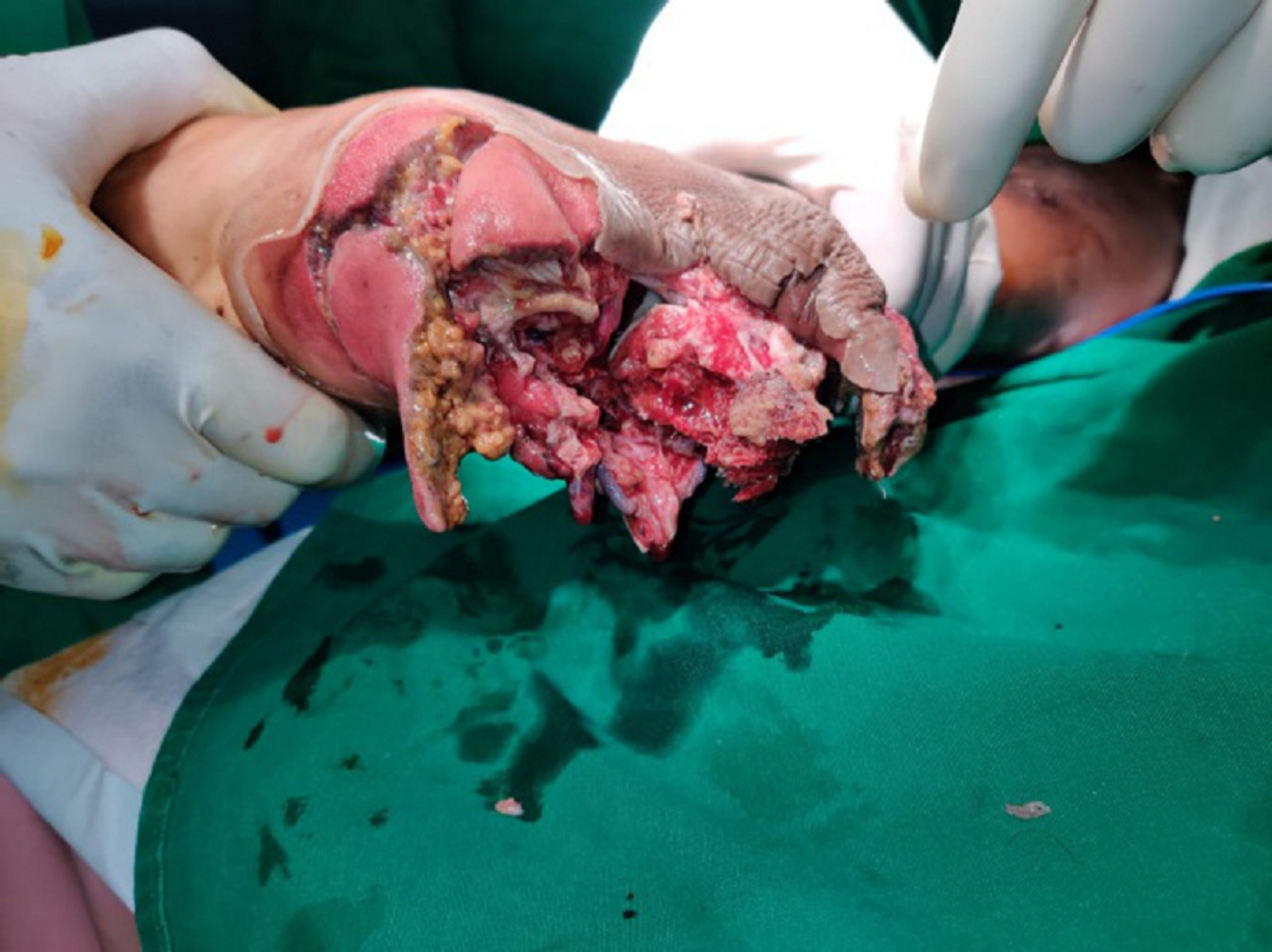
Crush injury of the right heel pad exposing the underlying soft tissue and bone.

A 22-year-old male patient was brought to R L Jalappa Hospital & Research Centre, affiliated with Sri Devaraj Urs Medical College, Kolar, India. The patient presented with an alleged history of a road traffic accident. He sustained a crush injury to his right heel pad, resulting in an open comminuted fracture of the right calcaneum with bone loss (Figure 1). The range of motion at the right ankle and the subtalar joint was painful and restricted. Dorsalis pedis artery pulsation was palpable, and distal sensation was intact. Active toe movements were present. The capillary refill time was normal. All other long bones and joints were clinically normal. There were no other injuries anywhere in the body.

**Figure 2:**
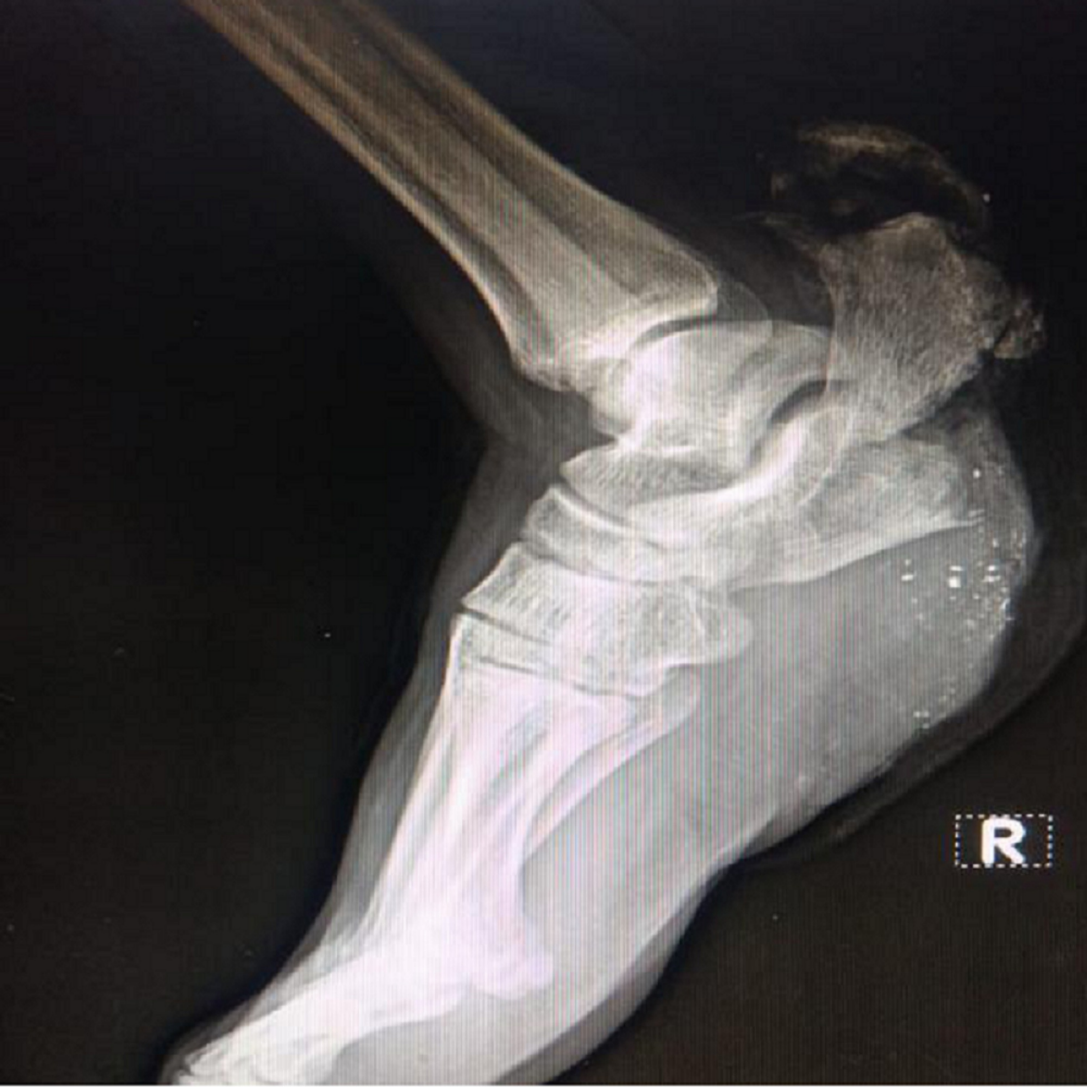
Plain radiographs of the right foot lateral view showing comminuted right calcaneum fracture with bone loss.

**Figure 3:**
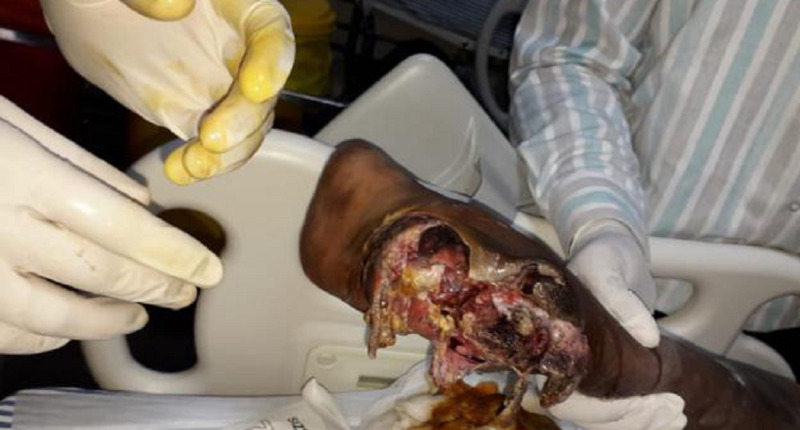
Clinical picture of the right heel pad after three days following initial debridement.

At the emergency department, triple antibiotic prophylaxis was administered. It consisted of intravenous (IV) administration of augmentin 1.2 g, amikacin 500 mg, and metronidazole 100 ml. Wound wash was given with six liters of sterile saline at the bedside and the wound site dressed with moist gauze. The patient was stabilized with a below-knee splint and sent for radiographs. Preoperative radiographs are provided in Figure 2. After imaging was completed and informed consent obtained, the patient underwent thorough wound debridement and NPWT was given. The initial operative encounter consisted of the patient being operated under spinal anesthesia. Gross wound contaminants were removed, including all devitalized bone. Three liters of sterile saline were then used to irrigate the wound. Once complete, all wounds were left open and a wound VAC was applied. Figure 3 shows the clinical picture of the right heel pad after three days following initial debridement.

Vacuum-assisted closure (VAC) technique

**Figure 4:**
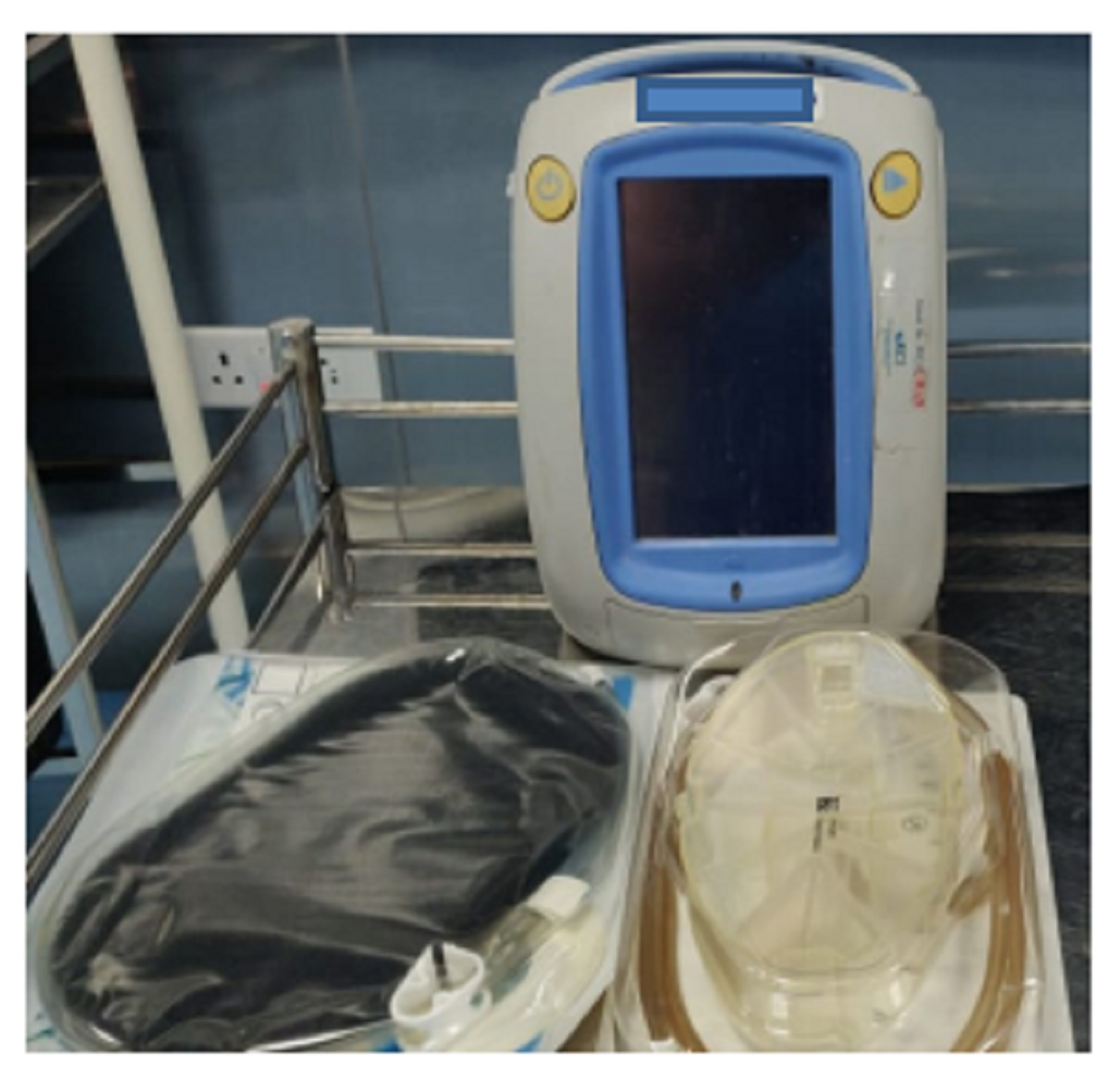
Vacuum-assisted closure (VAC) device

The wounds were then lightly packed with sponge and sealed with adhesive drapes. The sterile sponge was applied over the exposed part of the bone and hardware. When sealing the sponge, care was taken to lay drapes over the skin with minimal skin tension. Suction was set at -125 mm of Hg. A total of seven days of NPWT was allowed [2]. The vacuum-assisted closure device is shown in Figure 4.

Hospital course

**Figure 5:**
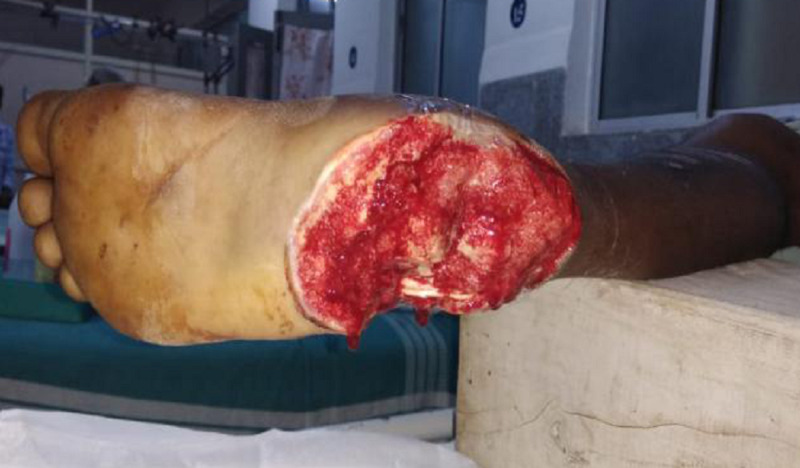
Clinical picture of the right heel pad after seven days of secondary wound debridement and with negative pressure wound therapy

**Figure 6:**
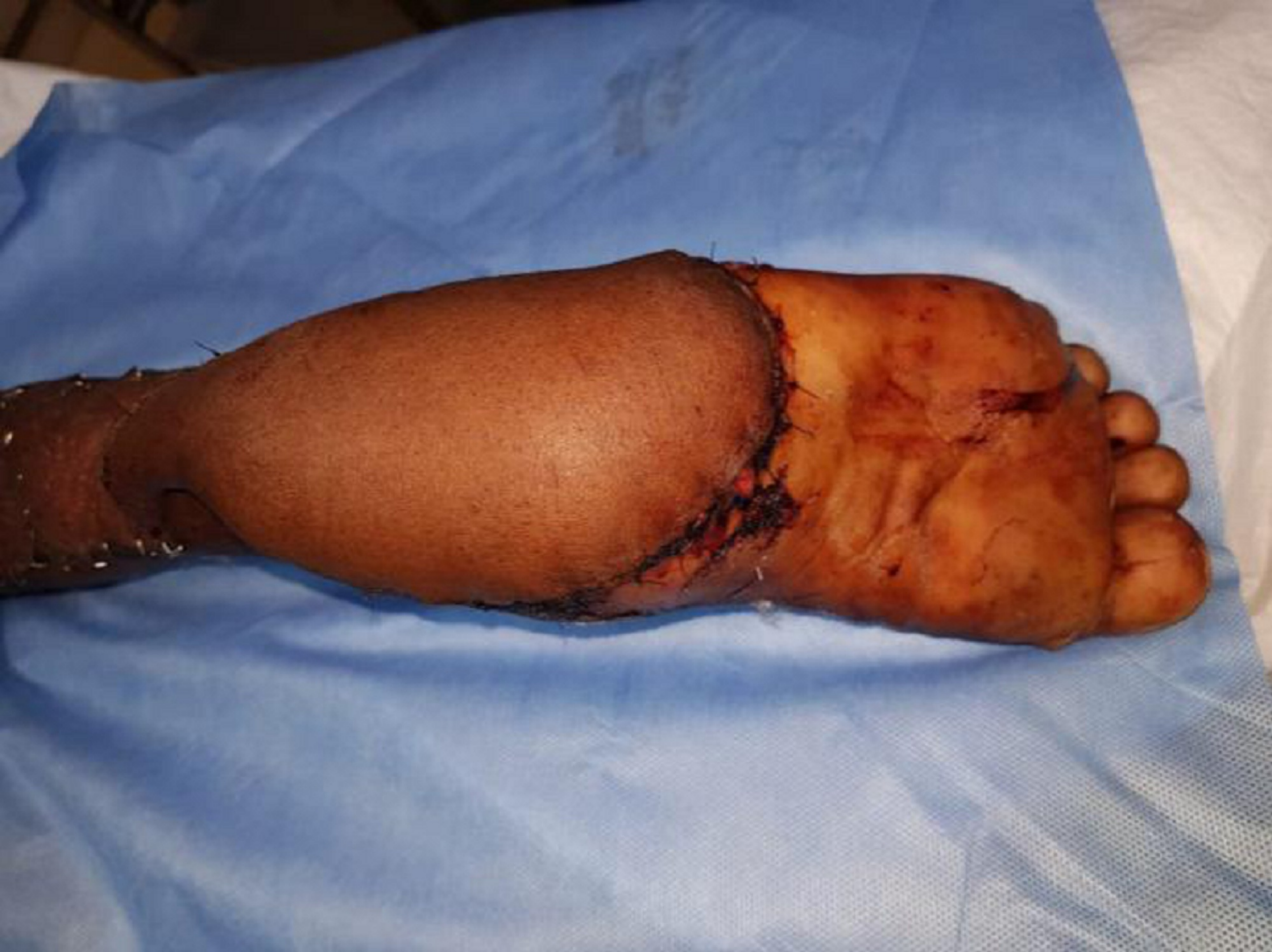
Clinical picture of the right heel pad reconstruction with reverse sural artery flap

**Figure 7:**
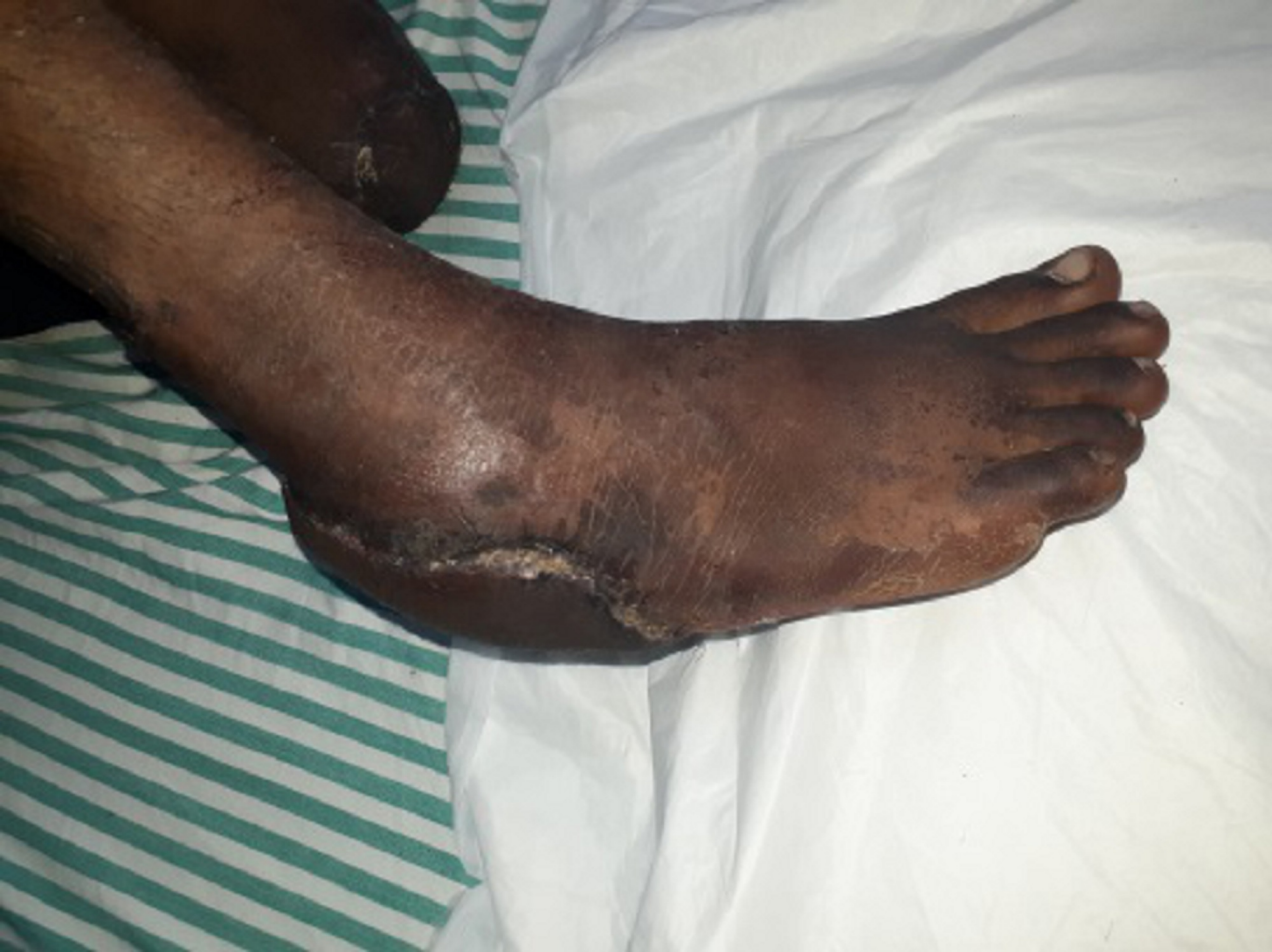
Clinical picture of the right heel pad lateral aspect at three months follow-up

**Figure 8:**
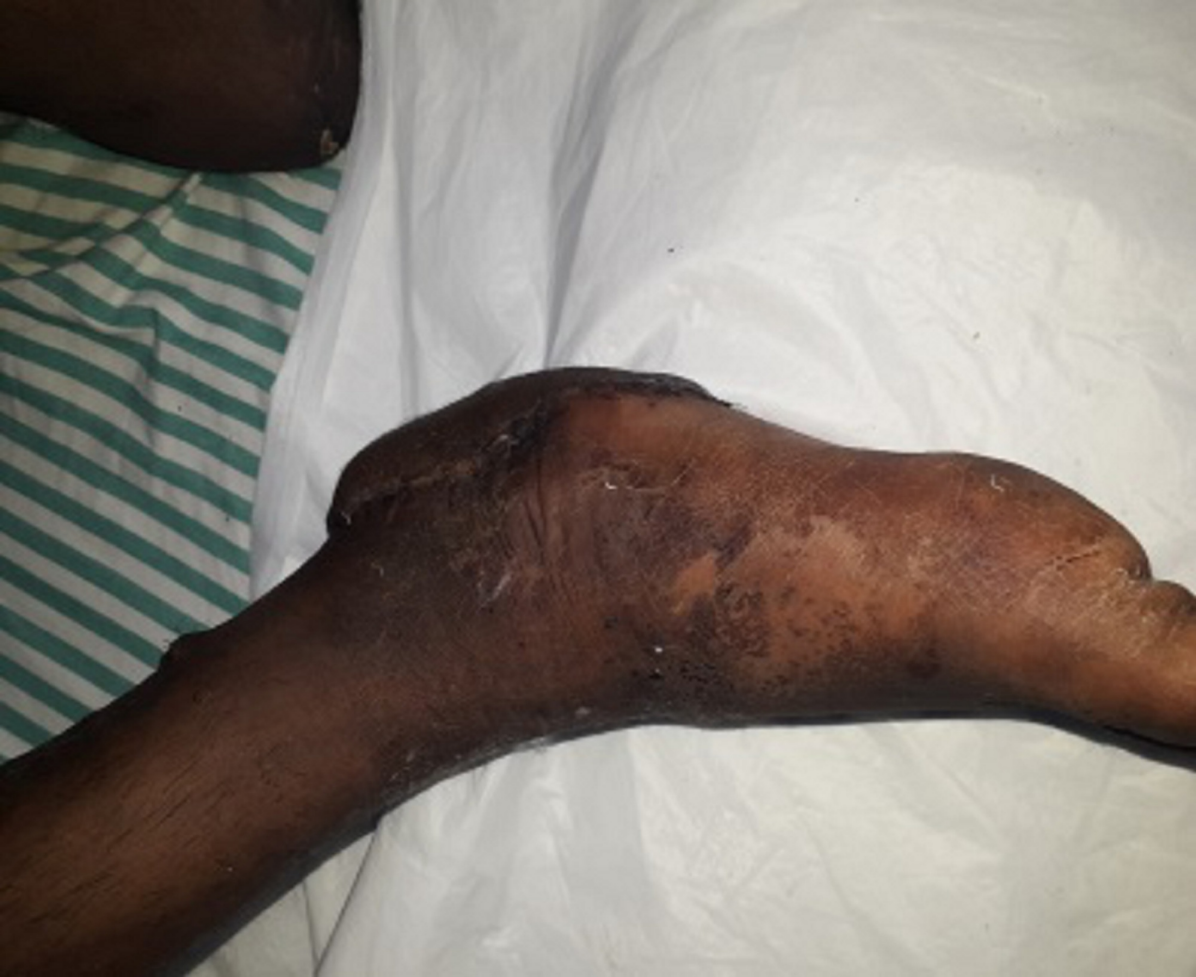
Clinical picture of the right heel pad medial aspect at three months follow-up

**Figure 9:**
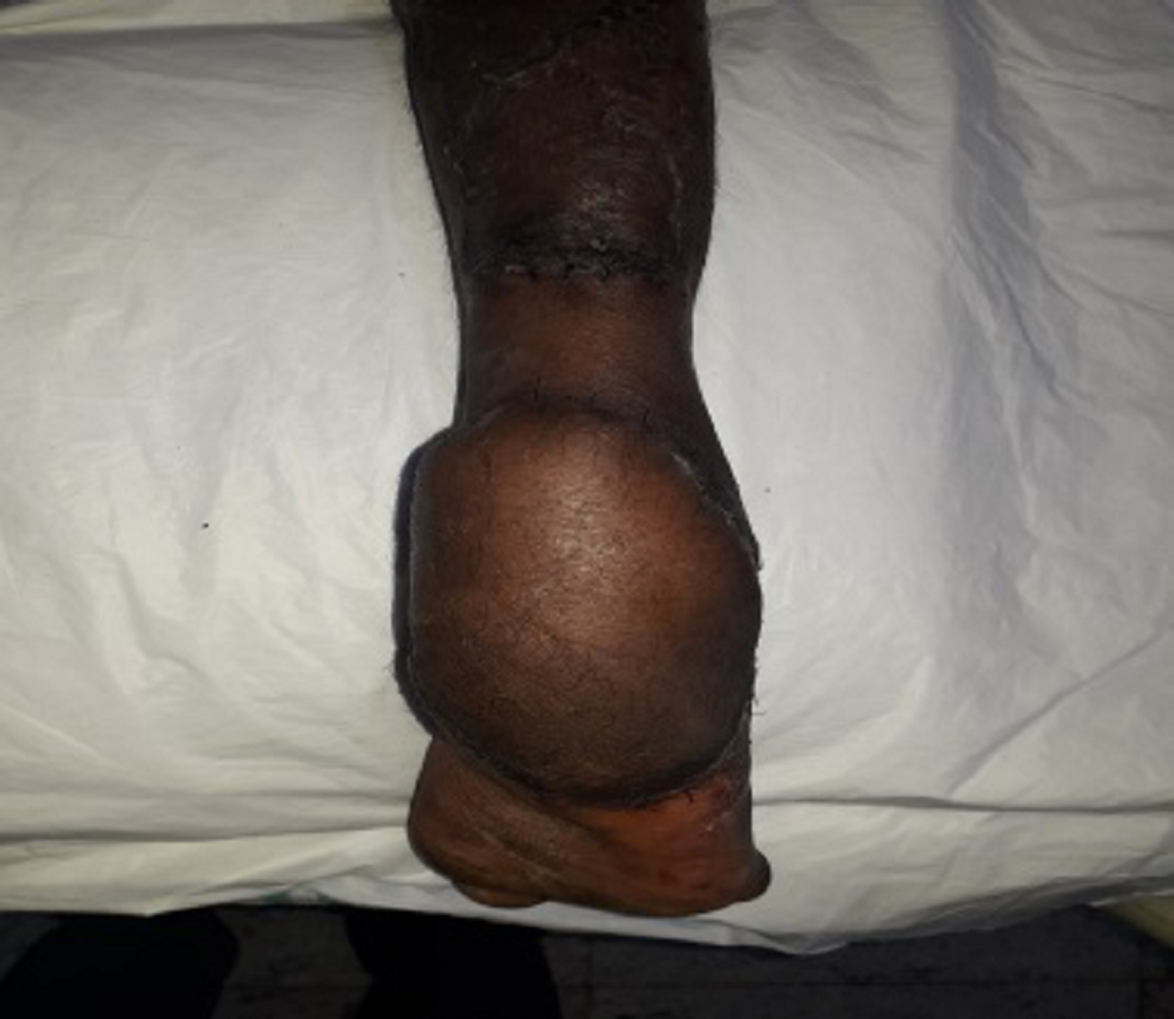
Clinical picture of the right heel pad reconstructed with reverse sural artery flap

**Figure 10:**
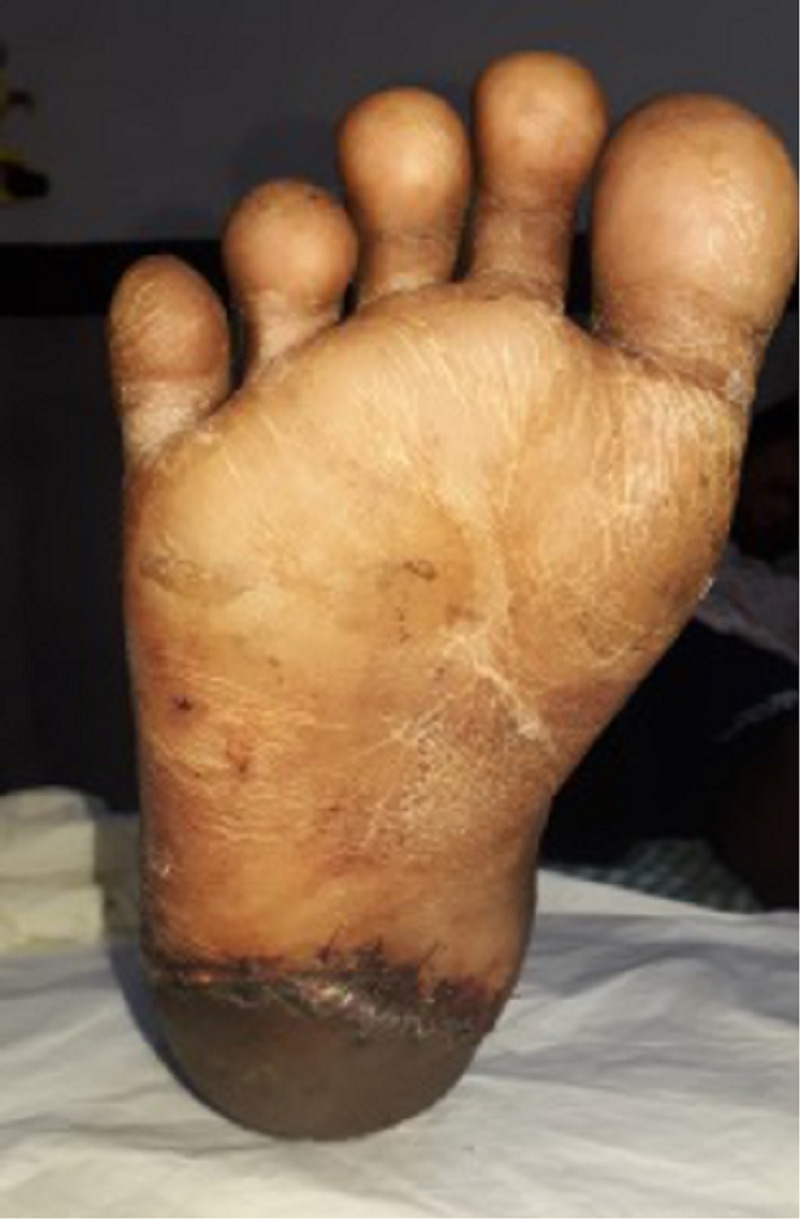
Clinical picture of the right heel pad plantar aspect at three months follow-up

Postoperatively, the patient received intravenous augmentin 1.2 g twice daily for seven days, amikacin 500 mg twice daily for seven days, and metronidazole 100 ml thrice daily for three days. It was followed by oral Amoxiclav 625 mg twice daily for seven days. The patient was taken to the operating room one more time for wound debridement (Figure 5), followed by reverse sural artery flap placement (Figure 6). Assessment of the wound during the time of secondary debridement demonstrated a progressive wound bed with granulation tissue and a decrease in overall wound size. Postoperatively, the patient stayed in the hospital for seven days for flap monitoring. The surgical site healed well (Figures 7-10). Active dorsiflexion of the right ankle was present. The patient is being followed at regular intervals. Outpatient follow-up ensued at two weeks, one month, and three months.

## Discussion

The VAC drape helps in providing a wound healing environment. VAC GranuFoam dressings contract under negative pressure. It provides direct and complete contact with the wound bed. The VAC canister collects the wound exudate. Four-hundred to 600 micron-reticulated pores help distribute pressure through the wound bed [3-4]. VAC GranuFoam dressing - Black is hydrophobic, and its open-pore nature (400-600 microns) provides a uniform application of negative pressure at the wound site. It helps in wound contraction. VAC - white foam dressing is hydrophilic, pre-moistened with sterile water, and is a denser foam with a greater pore size distribution. It requires a higher pressure of 125-175 mm of Hg to provide adequate negative pressure therapy distribution. The application of uniform negative pressure delivered by VAC therapy induces a physical response (macrostrain) and a biological response (microstrain). Macrostrain draws wound edges together and removes exudate and infectious material. It reduces edema, and promotes perfusion. Microstrain creates tissue micro deformation, causing cells to stretch [5]. Cell stretch leads to the migration of cells and proliferation that result in granulation tissue formation.

VAC therapy is indicated in acute wounds - traumatic, partial-thickness burns, flaps, and grafts, in subacute dehisced wounds, and in chronic conditions - diabetic ulcers, pressure injuries, and venous insufficiency ulcers. VAC therapy contraindications include foam dressing directly in contact with exposed blood vessels, anastomotic sites, organs or nerves, malignancy in the wound, non-enteric and unexplored fistulas, necrotic tissue with eschar present, sensitivity to silver (VAC GranuFoam silver dressing only), and untreated osteomyelitis.

Nicks B A et al. in a study proved the management of acute wounds varies based on location and characteristics [6]. A systematic approach gives the best framework for managing acute wounds. NPWT helps in providing temporary wound coverage during the period of debridement and closure of the wound.

Careful selection of the patient and the procedure, along with the appropriate technique, is imperative for NPWT success. The wound healing process involves coagulation, inflammation followed by a migratory and proliferative stage, and, finally, remodeling. NPWT is shown to increase cell proliferation, increase local perfusion, increase granulation, and clear wound infections earlier. It will decrease hospital length of stay, and expedite the final closure of the wound. Khurram M F et al. demonstrated that VAC therapy accelerates the healing process by granulation tissue formation and reduces morbidity and pain in pediatric patients [7]. The sizes of soft tissue defects, reduced from 69.18 cm^2^ to 50.73 cm^2^ after VAC in their study. Yadav S et al. proposed that this novel VAC technique helps in the management of difficult wounds [8]. It has always been a cause of concern for the treating clinicians and VAC delivers at par or better results as compared to the conventional techniques. Webster J et al. in their study urged the need for larger, well-designed, and well-conducted trials to evaluate the effects of NPWT for use on clean, closed surgical wounds [9]. The trials should initially focus on wounds that are difficult to heal like sternal wounds or incisions on obese patients. Huang C et al. in their study suggested that for better care of patients, it is essential to stay updated on advancements in mechanobiology, cell therapy, and biofilms [10]. The optimal parameters for specific wounds, including interface material, the waveform of suction application, and the amount of suction, are to be analyzed. The disadvantages of VAC are increased cost, additional technical requirements, and required inpatient monitoring of the system.

Treating patients with extensive soft tissue damage and high-grade compound fractures is challenging, as it requires a complex approach with plastic, orthopedic and vascular-reconstructive procedures. Management involves combinations of closure after wound debridement by secondary intention, VAC device usage, and various reconstructive plastic surgery methods. We authors noted that the benefits significantly outweigh the costs of the VAC system, making it an essential treatment option for patients similar to the one presented in this case report.

## Conclusions

The patient in our case report had better clinical and functional outcomes following NPWT for extensive soft tissue loss in the foot. NPWT is relatively a safer technique with less chance of infection than traditional dressing methods. Our hypothesis is that wound debridement followed by a single set of VAC placement for seven days should be tried for wound needing soft tissue coverage. Hence, it is a feasible and valuable method in the treatment of compound fractures with massive soft tissue defects.
